# Supplementary material for: Cats shedding pathogenic Leptospira spp.—An underestimated zoonotic risk?
Source: PLoS One. 2020 Oct 22;15(10):e0239991. doi: 10.1371/journal.pone.0239991 (PMC7580889; doi:10.1371/journal.pone.0239991)
Supplement: S1 Questionnaire — (DOCX) [file pone.0239991.s001.docx]

**Questionnaire**

| Date | Place | Name of the cat | ID (if hospital patient) | Owner name | Phone numer |
| --- | --- | --- | --- | --- | --- |
| Picture of the cat  Picture of the vaccination booklet | o taken  o taken | o does not exist |  |  |  |
| Vaccination | o no | o yes | name.......... | company ... | date |
| sex | o male | o female |  |  |  |
|  | o castrated or spayed | O intact |  |  |  |
| Age (in years) |  |  |  |  |  |
|  |  |  |  |  |  |
| breed |  |  |  |  |  |
|  |  |  |  |  |  |
| ***Risk factors***  Access to outside  Outside >50% of the time  Origin  Drinking out of puddles  Contact with rodents  Eating rodents  Eating raw meet | o yes  o yes  o urban  o yes  o yes  o yes  o yes | o no  o no  o rural  o no  o no  o no  o no |  |  |  |
| Status | o pet cat | o stray cat |  |  |  |
| Contact with other cats | o pet cats | o stray cats | o no |  |  |
| Contact with dogs | o pet dogs | o stray dogs | o no |  |  |
| Contact with livestock | o yes | o no |  |  |  |
