# Supplementary material for: Cats shedding pathogenic Leptospira spp.—An underestimated zoonotic risk?
Source: PLoS One. 2020 Oct 22;15(10):e0239991. doi: 10.1371/journal.pone.0239991 (PMC7580889; doi:10.1371/journal.pone.0239991)
Supplement: S2 Questionnaire — (DOCX) [file pone.0239991.s002.docx]

**Cuestionario**

| Fecha | Lugar | Nombre de gato: | Numero de ficha | Nombre de propriedario | Numero de telefono |
| --- | --- | --- | --- | --- | --- |
| Foto del animal  Foto del certificado de vacunación | o hecha  o hecha | o no existente |  |  |  |
| Vacuna | o no | o si | nombre .......... | ....... productor ... | ... fecha ...... |
| sexo | o macho | o hembra |  |  |  |
| estado | o castrado | O entero |  |  |  |
| edad (años) |  |  |  |  |  |
|  |  |  |  |  |  |
| raza |  |  |  |  |  |
|  |  |  |  |  |  |
| ***Factores de riesgo***  Corre libre  Está fuera de la casa >50% del tiempo  origen  suele beber de charcos  contacto con roedores  come roedores  consume carne cruda | o si  o si  o urbano  o si  o si  o si  o si | o no  o no  o rural  o no  o no  o no  o no |  |  |  |
| estado | o casero | o salvaje |  |  |  |
| Contacto con otros gatos | o casero | o salvaje | o no |  |  |
| Contacto con perros | o casero | o salvaje | o no |  |  |
| Contacto con ganado | o si | o no |  |  |  |
