# Supplementary material for: Cats shedding pathogenic Leptospira spp.—An underestimated zoonotic risk?
Source: PLoS One. 2020 Oct 22;15(10):e0239991. doi: 10.1371/journal.pone.0239991 (PMC7580889; doi:10.1371/journal.pone.0239991)
Supplement: Attachment — Submitted filename: Andrea Murillo-Review.docx [file pone.0239991.s003.docx]

It's a great job and it's been fun reviewing it.

Throughout the document, it would be more suitable to use the term: leptospires or *Leptospira spp.* Please remember that *Leptospira spp.* and gene *lipL*32 must be italicized*.*

**INTRODUCTION**

It might be helpful for the readers, to briefly introduce the diagnostic techniques employed.

Line 26: Even considering the findings in reference 19? Please rejigging the sentence.

55-57: A reference is needed.

63-64: The statement, is contrary to the high seroprevalence of 48.5%. I haven't read any reports with such a high value of 48.5% in cats. None of the references you mention report those values. Is it possible that a reference has been missed or is it a typing accident?.

71: Please verify reference 18, as they did not perform PCR.

**MATERIAL AND METHODS**

110: Considering table 2, I think the cats were cats that attended the veterinary service and not shelters or spay programs, right? maybe it is better to highlight it here.

124-127: Check the wording

151: Do you have a reference on which you based the PCR methodology? It is important to outline species and strain from the positive control, limits of detection and if you performed replicates or duplicates?

157: There is missing information, reference 20 did not use IMS method. Do you have a reference?

168: The liquid media was supplemented with something? i.e. bovine serum albumin, 5-Fluorouracil, rabbit serum.

198: Thaipadungpanit, et al. is not referenced, so it's not possible to follow the protocol. Please reference them.

225: Report the *P-*value for the conditional model.

225: Please refer also to the authors of the lme4 package and R core team in references.

I have a question about culture coupled-IMS. Was the urine used for IMS diluted with PBS at the time of sampling? and if so, in what dilution? I understand from the methodology, it was added after, at the time of centrifugation. But during the time between sampling and the IMS, no. I've seen figure 1 but I'm still in doubt. How it might affect the viability of leptospires?

**RESULTS**

278: It would be easier for readers if in table 5,6 and 7 is included a column with the OR *P-*value.

280: I wonder about the *P-*value of contact with other cats and dogs? The OR is close to or greater than 1.

309: as for urinary shedding (Table 6) ,P-value here is <0.05?

**DISCUSSION**

350: Please reference the study to give the correct weight to the statement

352: What about cat’s urine pH?

371-372: This is a good place to explain and consider the demanding growing conditions for *L. interrogans* serovar Copenhageni.

368: It would be interesting to discuss the *Leptospira* spp. found in these cats against those found in other studies in cats.

394-406: I agree with you. Considering that you have some clinical information about these cats, you can focus this part of the discussion on sick cats that shed leptospires, distinguish them as acute or chronic sick/infected, because those are two states of infection that are different.

408: Rats are reservoirs of the Icterohaemorrhagiae and Copenhageni serovars.

Please include in the discussion information about why the positives to a single culture were not positives to IMS-culture and/or vice versa?
