# Supplementary material for: Cats shedding pathogenic Leptospira spp.—An underestimated zoonotic risk?
Source: PLoS One. 2020 Oct 22;15(10):e0239991. doi: 10.1371/journal.pone.0239991 (PMC7580889; doi:10.1371/journal.pone.0239991)
Supplement: Attachment — Submitted filename: Feedback for Plos one .docx [file pone.0239991.s004.docx]

The purposes of this study were to determine the proportion of cats shedding pathogenic Leptospira in Southern Chile, to determine the molecular profile of the cultured isolates, and to identify possible risk factors regarding *Leptospira* spp. shedding.

I think the results of this paper are definitely very interesting and highlight the importance of further investigating leptospirosis in cats. However, there are still some issues need to be addressed before further consideration.

Abstract:

Line 25: Change the Leptospira to Leptospira spp.

Line 26: This sentence need to be rephrased as in the reference 19, it has been reported from urine and kidney.

Line 33: Immunomagnetic

Introduction:

Line 59: should be under investigated rather than rarely suffer.

Line 63: The proportions were also very much depending on samples size and laboratory techniques used. Prevalence is more commonly used. Suggest changing the proportions to prevalence.

Line 69-76: Suggest shortening the paragraph.

Line 84-86: This was the result obtained from previous publication, it will be more informative if authors can share about the importance of that finding.

Line 87: How big is the human population? Since this paper concerns about the role of cats in leptospirosis. Strongly suggest the authors to add in human leptospirosis in Chile in the introduction in order to highlight how severe is the problem in human and lead to the investigating in companion animal.

Material and methods:

Line 104: Does the locations and time of sampling affected the result?

Line 111: would suggest providing a justification for the inclusion for outdoor cat only in discussion part. The definition of outdoor need to be specified. Roam freely? Community cat?

Line 117: The physical examination was performed by who? Was there any consent from owner?

Line 124: MAT is always the gold standard in investigating leptospirosis. Is there any reason why it was not performed?

Line 123: There is lack of justification the usage of qPCR, IMS-qPCR, culture and IMS-coupled culture concurrently in this study. From the line 336 to 346, IMS-qPCR was reported to be more sensitive than qPCR from the previous study. Hence, is the comparison between the different techniques also one of the objectives of the study?

Table 2

Please be more specific about the chronically ill. Would like to know what type of illness.

Discussion:

Line 332: The recruited cats were not indoor, hence, how accurate the owner can observe about the rat-hunting and eating behaviour?

Line 349-351: Since it has been reported before, I will recommend removing “first one”.

Line 355-356: Need to discuss a little bit about the findings obtained from direct culture and IMS-coupled culture. Why there were certain samples positive with direct culture, some with IMS-coupled culture?

Line 424-425: it also affected by the stage pf the leptospirosis

Line 427: that should be one of the aims of the study
