# Supplementary material for: Cats shedding pathogenic Leptospira spp.—An underestimated zoonotic risk?
Source: PLoS One. 2020 Oct 22;15(10):e0239991. doi: 10.1371/journal.pone.0239991 (PMC7580889; doi:10.1371/journal.pone.0239991)
Supplement: Attachment — Submitted filename: Response to reviewers (1).docx [file pone.0239991.s005.docx]

# **Response to Editor and Reviewers:**

Dear Editor, dear Reviewers,

thank you very much for appreciating the importance of our work and for giving us the opportunity to further improve our manuscript. We carefully addressed all comments and questions and subsequently revised the manuscript to the reviewers‘ suggestions. The cited line numbers refer to changes in the revised manuscript without track changes.

# **Editor comments**

Dear Dr. Dorsch,
Thank you for submitting your manuscript to PLOS ONE. After careful consideration, we feel that it has merit but does not fully meet PLOS ONE’s publication criteria as it currently stands. Therefore, we invite you to submit a revised version of the manuscript that addresses the points raised during the review process.
==============================
ACADEMIC EDITOR: Estimation of shedding load formula need appropriate reference support. Information about the pH of the urine sample give some clue to improvise the isolation methods.

Thank you for these comments.

Respective references for the shedding load formula were added.

Please see new line 208:

Tomckowiack C, Henriquez C, Ramirez-Reveco A, Muñoz P_,_ Collado B, Herzberg D, et al. Analytical evaluation of an immunomagnetic separation-PCR assay to detect pathogenic *Leptospira* in cattle urine samples obtained under field conditions. J Vet Diagn Investig. 2020 (accepted for publication July 8th 2020)

Dzieciol M, Volgger P, Khol J, Baumgartner W, Wagner M, Hein I. A novel real-time PCR assay for specific detection and quantification of Mycobacterium avium subsp. paratuberculosis in milk with the inherent possibility of differentiation between viable and dead cells. BMC research notes. 2010;3:251. doi: 10.1186/1756-0500-3-251.

We measured USG and urine pH in most of the samples. However, in the present study, there was no significant difference in urine pH and urine specific gravity (USG) between PCR-positive (mean pH 6.7, mean USG 1.044) and PCR-negative cats (mean urine pH 6.7, mean USG 1.040) (P>0.05), and between culture-positive (mean pH 6.3, mean USG 1.047) and culture-negative cats (mean urine pH 6.7, mean USG 1.043) (P>0.05).However, we have to be cautious in the sense that these were 220 culture-negative against 7 culture-positive individuals

We added a new paragraph in the discussion and 2 new references.

Please see new line 364-368:

“Low pH is another physicochemical property of the urine contributing to the defense system against bacteria (Smee et al., 2013), and *Leptospira* spp. do not survive well in acidic urine but remain viable in alkaline urine (Levett 2001). However, in the present study, the mean urine pH was identical in culture-positive and in culture-negative cats.”

Smee N, Loyd K, Grauer G. UTIs in small animal patients: Part 1: Etiology and pathogenesis. J Am Anim Hosp Assoc 2013;49:1–7.

Levett PN. Leptospirosis. Clin. Microbiol. Rev. 2001,14(2):296. DOI: 10.1128/CMR.14.2.296-326.2001.

**Journal Requirements:**

When submitting your revision, we need you to address these additional requirements.

1. Please ensure that your manuscript meets PLOS ONE's style requirements, including those for file naming. The PLOS ONEstyle templates can be found at

<https://journals.plos.org/plosone/s/file?id=wjVg/PLOSOne_formatting_sample_main_body.pdf> and <https://journals.plos.org/plosone/s/file?id=ba62/PLOSOne_formatting_sample_title_authors_affiliations.pdf>

Sorry for that. The file naming was adapted to fit the journal´s requirements.

2. In your Methods section, please provide additional details regarding participant consent from the owners of the animals. In the ethics statement in the Methods and online submission information, please ensure that you have specified (1) whether consent was informed and (2) what type you obtained (for instance, written or verbal). If the need for consent was waived by the ethics committee, please include this information.

Informed written owner consent was obtained for all animals included in the study. This information was added in the online submission information and also in the manuscript.

Please see new line 108:

3. Please include additional information regarding the survey or questionnaire used in the study and ensure that you have provided sufficient details that others could replicate the analyses. For instance, if you developed a questionnaire as part of this study and it is not under a copyright more restrictive than CC-BY, please include a copy, in both the original language and English, as Supporting Information.

We attached the questionnaire used in the present study in Spanish (original language) and in English as Supporting Information.

4. In your Methods section, please state where the participants were recruited for your study.

Included cats were either patients of the small animal clinic of the Universidad Austral de Chile in Valdivia, presented for castration or spaying during castration initiatives of the city of Valdivia, or were privately owned cats with owners signing up for participation in the study. Respective information was added.

Please see new lines 119-127:

“Included cats were patients of the small animal hospital of the Universidad Austral de Chile in Valdivia presented for health issues or for elective procedures (e.g. neutering, vaccination), client-owned cats participating in castration projects offered by the city of Valdivia or other communities, and and cats presented by their owners specifically for study enrolement. Cats were offered one free vaccination and deworming as compensation for participating, if this was clinically justifiable. Cats were excluded from the study if they had received antimicrobial treatment within the last four weeks before sampling due to a decreased likelihood for detection of *Leptospira* spp. even if they were infected, or if they were living exclusively indoors or had only access to a balcony because of a low risk for infection.”

”

5.We note that [Figure(s) 2] in your submission contain [map/satellite] images which may be copyrighted. All PLOS content is published under the Creative Commons Attribution License (CC BY 4.0), which means that the manuscript, images, and Supporting Information files will be freely available online, and any third party is permitted to access, download, copy, distribute, and use these materials in any way, even commercially, with proper attribution. For these reasons, we cannot publish previously copyrighted maps or satellite images created using proprietary data, such as Google software (Google Maps, Street View, and Earth). For more information, see our copyright guidelines: <http://journals.plos.org/plosone/s/licenses-and-copyright>.

Figure 2 was drawn by one of the co-authors (Theo Eberhard). Therefore, it is not copyrighted.

6.Thank you for stating the following financial disclosure:

Bavarian Academic Center for Latin America supported the study by paying for travel expenses (between Valdivia and Munich on two occasions) for planing the study. The Dirección de Investigación y Desarollo of the Universidad Austral de Chile supported the study by covering part of the costs for the material necessary for sampling and part of additional costs, e.g. transport and compensation of owners for study participation. For compensation, all included cats were vaccinated and dewormed. MSD Animal health provided the resources for sampling, clinical investigation of cats and all laboratory investigations. H.L.B.M. Klaasen is an employee of a company (MSD Animal Health) developing and marketing animal vaccines and pharmaceuticals. He contributed in preparation of the the mansucript and had valuable input in interpretation of the data.

Please state what role the funders took in the study.  If the funders had no role, please state: "The funders had no role in study design, data collection and analysis, decision to publish, or preparation of the manuscript."

If this statement is not correct you must amend it as needed.

The statement was amended accordingly. We added also, that part of the lab work was funded by Proyecto FIC 15-8 (código BIP 30421496) del Gobierno Regional de Los Ríos, Chile.

It now reads:

“The Bavarian Academic Center for Latin America supported the study by paying for travel expenses (between Valdivia and Munich on two occasions) for planning the study. The Dirección de Investigación y Desarollo of the Universidad Austral de Chile supported the study by covering part of the costs for the material necessary for sampling and part of additional costs, e.g. transport and compensation of owners for study participation. For compensation, all included cats were vaccinated and dewormed. MSD Animal health provided the resources for sampling, clinical investigation of cats and all laboratory investigations. Part of the laboratory work was funded by Proyecto FIC 15-8 (código BIP 30421496) del Gobierno Regional de Los Ríos, Chile.

H.L.B.M. Klaasen is an employee of a company (MSD Animal Health) developing and marketing animal vaccines and pharmaceuticals. He contributed in preparation of the manuscript and had valuable input in interpretation of the data. The funders had no role in study design, data collection and analysis and decision to publish. The contribution of H.L.B.M Klaasen (being a senior scientist working in a research department) in preparation of the manuscript was purely a scientific one, mainly related to his knowledge of and experience with leptospirosis research.”

Please include this amended Role of Funder statement in your cover letter; we will change the online submission form on your behalf.

We included the amended Role of Funder statement in our cover letter.

7.Thank you for stating the following in the Competing Interests section:

H.L.B.M. Klaasen is an employee of a company (MSD Animal Health) developing and marketing animal vaccines and pharmaceuticals. Otherwise the authors declare that they have no conflict of interest with respect to the research, authorship and/or publication of this article.

Please confirm that this does not alter your adherence to all PLOS ONE policies on sharing data and materials, by including the following statement: "” (as detailed online in our guide for authors <http://journals.plos.org/plosone/s/competing-interests>).  If there are restrictions on sharing of data and/or materials, please state these. Please note that we cannot proceed with consideration of your article until this information has been declared.

Please include your updated Competing Interests statement in your cover letter; we will change the online submission form on your behalf.

We included an updated Competing Interest Statement in our cover letter.

“H.L.B.M. Klaasen is an employee of a company (MSD Animal Health) developing and marketing animal vaccines and pharmaceuticals. Otherwise the authors declare that they have no conflict of interest with respect to the research, authorship and/or publication of this article. The contribution of H.L.B.M Klaasen (being a senior scientist working in a research department) was purely a scientific one, mainly related to his knowledge of and experience with leptospirosis research. This does not alter our adherence to PLOS ONE policies on sharing data and materials.”

Please know it is PLOS ONE policy for corresponding authors to declare, on behalf of all authors, all potential competing interests for the purposes of transparency. PLOS defines a competing interest as anything that interferes with, or could reasonably be perceived as interfering with, the full and objective presentation, peer review, editorial decision-making, or publication of research or non-research articles submitted to one of the journals. Competing interests can be financial or non-financial, professional, or personal. Competing interests can arise in relationship to an organization or another person. Please follow this link to our website for more details on competing interests: [http://journals.plos.org/plosone/s/competing-interests](about:blank)

**Additional Editor Comments (if provided):**

The qPCR technique using LipL32 need the reference support to know the probe details in the method section.

qPCR was performed as previously descriped by Stoddard et al. (2009) The respective reference has been included.

Please see new line 159:

Stoddard RA, Gee JE, Wilkins PP, McCaustland K, Hoffmaster AR. Detection of pathogenic Leptospira spp. through TaqMan polymerase chain reaction targeting the LipL32 gene. Diagn Microb Inf Dis. 2009;64(3):247-55. doi: 10.1016/j.diagmicrobio.2009.03.014.

Really wonder to notice that out of the 7 culture/IM culture positive cases just one turned to be positive for PCR and the justification of presence of PCR inhibitors in the urine need more authentication. If it is the case, then it reflects on the negative samples as false negatives due to the PCR inhibitors. Since they have used the high pure PCR template preparation kit and practically, they give high pure genomic DNA without any inhibitors. Authors need to explain it well in the discussion.

The DNA extraction-purification protocol using the High Pure PCR Template Preparation kit (Roche) is not specifically intended for urine/fecal samples, as there are some others, such as Qiagen’s stool kit (which are much more expensive), and that also use InhibitEX tablet contained in the kit that are necessary for total removal of inhibitors. So, for this reason it was discussed that PCR-inhibitors could still be present in the DNA extracted with the used method. Probably, some ions, reported to inhibit PCR by competition with Mg2+ ions (Bickley et al., 1996), stayed in the water phase throughout the process. To remedy this, the urine pellet resulting from initial centrifugation was washed to reduce Ca2+ content as well as many other inhibitors, but we cannot assure total elimination.

Interestingly, a similar discrepancy was seen in a recent study in cats reporting the isolation of Leptospira spp. from feline kidneys and urine (Alashraf et al., 2020). In that study, all 4 culture positive cats were PCR-negative. This repeated pattern in studies by different researchers is a strong indication that there are substances/chemicals used in the PCR processing (and probably not in the samples that are cultured) that are inhibitory for certain steps in the PCR.

Bickley, J., Short, J.K., McDowell, D.G. & Parkes, H.C. (1996). Polymerase chain reaction

(PCR) detection of Listeria monocytogenes in diluted milk and reversal of PCR inhibition

caused by calcium ions. Lett Appl Microbiol 22(2), 153-8.

Alashraf AR, Lau SF, Khairani-Bejo S, Khor KH, Ajat M, Radzi R, et al. First report of pathogenic Leptospira spp. isolated from urine and kidneys of naturally infected cats. PLoS One. 2020;15(3):e0230048. doi: 10.1371/journal.pone.0230048.

We added a new paragraph in the discussion. Please see new lines 381-385:

“Interestingly, a similar discrepancy was seen in the only other study in cats reporting the isolation of *Leptospira* spp. from feline kidneys and urine (Alashraf et al., 2020). In that study, all 4 culture-positive cats had negative urine PCR results. This indicates that there might be substances/chemicals in the PCR processing and not in the samples that are cultured that are inhibitory for certain steps in the PCR.”

Estimation of shedding load formula need appropriate reference support.

The respective references have been added.

Dzieciol M, Volgger P, Khol J, Baumgartner W, Wagner M, Hein I. A novel real-time PCR assay for specific detection and quantification of Mycobacterium avium subsp. paratuberculosis in milk with the inherent possibility of differentiation between viable and dead cells. BMC research notes. 2010;3:251. doi: 10.1186/1756-0500-3-251.

Tomckowiack C, Henriquez C, Ramirez-Reveco A, Muñoz P_,_ Collado B, Herzberg D, Folch H, Salgado M. 2020. Analytical evaluation of an immunomagnetic separation-PCR assay to detect pathogenic *Leptospira* in cattle urine samples obtained under field conditions. J Vet Diagn Investig. 2020 (accepted for publication July 8th 2020)

Is there any information about the pH of the collected urine sample. That would give some idea to improvise the isolation frequency.

We measured USG and urine pH in most of the samples. However, in the present study, there was no significant difference in urine pH and urine specific gravity (USG) between PCR-positive (mean pH 6.7, mean USG 1.044) and PCR-negative cats (mean urine pH 6.7, mean USG 1.040) (P>0.05), and between culture-positive (mean pH 6.3, mean USG 1.047) and culture-negative cats (mean urine pH 6.7, mean USG 1.043) (P>0.05).However, we have to be cautious in the sense that these were 220 culture-negative against 7 culture-positive individuals

We added a new paragraph in the discussion and 2 new references. Please see new line 364-368:

“Low pH is another physicochemical property of the urine contributing to the defense system against bacteria (Smee et al., 2013), and *Leptospira* spp. do not survive well in acidic urine but remain viable in alkaline urine (Levett 2001). However, in the present study, the mean urine pH was identical in culture-positive and in culture-negative cats.”

Smee N, Loyd K, Grauer G. UTIs in small animal patients: Part 1: Etiology and pathogenesis. J Am Anim Hosp Assoc 2013;49:1–7.

Levett PN. Leptospirosis. Clin. Microbiol. Rev. 2001,14(2):296. DOI: 10.1128/CMR.14.2.296-326.2001.

Reviewer #1:

It is a well-structured document with scientific rigour. The description of the methodology should be improved, for another researcher to reproduce the experiments described.

For details, please see the attached document. It's a great job and it's been fun reviewing it.

Throughout the document, it would be more suitable to use the term: leptospires or *Leptospira spp.* Please remember that *Leptospira spp.* and gene *lipL*32 must be italicized*.*

Thank you for this comment. The manuscript was checked for consistent use of “*Leptospira* spp.” and used italic type for this term as well as for the gene “*lipL32”.*

**INTRODUCTION**

It might be helpful for the readers, to briefly introduce the diagnostic techniques employed.

Line 26: Even considering the findings in reference 19? Please rejigging the sentence.

We rephrased this sentence accordingly. Now reads (Lines 24-26):

“Shedding of DNA of pathogenic *Leptospira* spp. has been documented in naturally infected cats in several countries, but urinary shedding of infectious *Leptospira* spp. has only recently been proven.”

55-57: A reference is needed.

We added a new reference. Please see new line 56:

Boey K, Shiokawa K, Rajeev S. Leptospira infection in rats: A literature review of global prevalence and distribution. PLoS Negl Trop Dis. 2019;13(8):e0007499. doi: 10.1371/journal.pntd.0007499.

63-64: The statement, is contrary to the high seroprevalence of 48.5%. I haven't read any reports with such a high value of 48.5% in cats. None of the references you mention report those values. Is it possible that a reference has been missed or is it a typing accident?.

Thank you very much for this comment. I am sorry for this mistake. This high number is from a doctoral thesis from France (Luciani 2004). As we were not able to get access to the original study, we later decided to take out this number and also the reference out of the manuscript. We changed the sentence accordingly.

Now reads (Lines 61-62):

“Reported antibody prevalence in naturally infected cats ranges from 0.8% to 33.3%, depending on the geographic area and the cats´ lifestyle [12-15].”

71: Please verify reference 18, as they did not perform PCR.

Thank you for this comment. We are sorry for this. The study of Larsson et al. (1986) was published only one year after invention of the PCR technique. The authors aimed to isolate leptospires from blood, urine and kidney tissue after cats were experimentally inoculated with a pathogenic and autochthonous field isolate of *Leptospira i*nterrogans and investigated the urine for leptospiral agglutinins but PCR was not performed. We rephrased the sentence accordingly.

Now reads (Lines 70-71):

“Few studies demonstrated shedding of *Leptospira* spp. in cats, first by identification of leptospiral agglutinins in the urine [19] and later by PCR [11, 12, 14-16, 20].”

**MATERIAL AND METHODS**

110: Considering table 2, I think the cats were cats that attended the veterinary service and not shelters or spay programs, right? maybe it is better to highlight it here.

Thank you for this comment. The cats were privately owned and there were no shelter cats included. The cats were patients of the small animal hospital presented for health issues or for elective procedures (spay, vaccination), client or community owned cats participating in castration projects offered by the city of Valdivia and cats of owners who volunteered for participation. This also included some recently adopted stray cats. Cats were offered one free vaccination and deworming as compensation if cats were healthy enough. Therefore, after promotion of our project, some owners were interested in bringing their cat to the clinic for study participation only, or owners were offered visits at their homes for sampling. We included a new paragraph in the material and methods section.

Now reads (Lines 119-127:):

“Included cats were patients of the small animal hospital of the Universidad Austral de Chile in Valdivia presented for health issues or for elective procedures (e.g. neutering, vaccination), client-owned cats participating in castration projects offered by the city of Valdivia or other communities, and and cats presented by their owners specifically for study enrolement. Cats were offered one free vaccination and deworming as compensation for participating, if this was clinically justifiable. Cats were excluded from the study if they had received antimicrobial treatment within the last four weeks before sampling due to a decreased likelihood for detection of *Leptospira* spp. even if they were infected, or if they were living exclusively indoors or had only access to a balcony because of a low risk for infection.”

124-127: Check the wording

It was re-worded. Now reads Please see new lines 139-142:

"To increase the overall diagnostic sensitivity for the detection of pathogenic *Leptospira* spp. in urine, four diagnostic tools were used in each sample: *lipL32* quantitative qPCR, immunomagnetic-separation (IMS)-coupled with *lipL32* qPCR (IMS-qPCR), culture for *Leptospira* spp., and IMS-coupled with culture (Fig 1).”

151: Do you have a reference on which you based the PCR methodology? It is important to outline species and strain from the positive control, limits of detection and if you performed replicates or duplicates?

The PCR was performed as described by Stoddard et al., 2009.

The respective reference has been added. Please see line 159:

Stoddard RA, Gee JE, Wilkins PP, McCaustland K, Hoffmaster AR. Detection of pathogenic Leptospira spp. through TaqMan polymerase chain reaction targeting the LipL32 gene. Diagn Microb Inf Dis. 2009; 64(3):247-55. doi: 10.1016/j.diagmicrobio.2009.03.014.

157: There is missing information, reference 20 did not use IMS method. Do you have a reference?

Sorry for that, this was a wrong reference and was removed.

168: The liquid media was supplemented with something? i.e. bovine serum albumin, 5-Fluorouracil, rabbit serum.

The EMJH liquid medium was supplemented with 5-fluorouracil for a final concentration of 0.2 mg/ml, but not with rabbit serum or bovine albumin or anything else.

Please see new lines 185-187:

“Two dilutions of urine (1:10 and 1:100) with 5 ml of liquid Ellinghausen-McCullough-Johnson-Harris (EMJH) medium supplemeted with 5-fluorouracil (0.2 mg/ml) were prepared immediately after urine sampling.”

198: Thaipadungpanit, et al. is not referenced, so it's not possible to follow the protocol. Please reference them.

The respective reference is included in the list of references now (reference 31):

Please see new line 617:

Thaipadungpanit J, Wuthiekanun V, Chierakul W, Smythe LD, Petkanchanapong W, Limpaiboon R, et al. A dominant clone of Leptospira interrogans associated with an outbreak of human leptospirosis in Thailand. PLoS Negl Trop Dis. 2007;1(1):e56. doi: 10.1371/journal.pntd.0000056.

225: Report the *P-*value for the conditional model.

The P-value is reported now.

Please see new lines 236-238:

“Variables associated with the outcome (P <0.25) were eligible for inclusion in the conditional model that was built using a forward approach and a P value of 0.05 was used for interpreting the outcomes.”

225: Please refer also to the authors of the lme4 package and R core team in references.

Respective references are included now (references 32 -34)

Please see new lines 622-629:

Bates D, Mächler M, Bolker B, Walker S (2015). “Fitting Linear Mixed-Effects Models Using lme4.” Journal of Statistical Software, **67**(1), 1–48. doi:[10.18637/jss.v067.i01](https://doi.org/10.18637/jss.v067.i01).

R Development Core Team (2008). R: A language and environment for statistical computing. R Foundation for Statistical Computing, Vienna, Austria. ISBN 3-900051-07-0, URL <http://www.R-project.org.>

I have a question about culture coupled-IMS. Was the urine used for IMS diluted with PBS at the time of sampling? and if so, in what dilution? I understand from the methodology, it was added after, at the time of centrifugation. But during the time between sampling and the IMS, no. I've seen figure 1 but I'm still in doubt. How it might affect the viability of leptospires?

Fresh native urine, not diluted with PBS, was used for IMS-coupled PCR and also for IMS-coupled with culture. The time window was typically 2 to 3 hours. In the laboratory, the urine sample was subjected to a purification protocol that consisted of a double centrifugation followed by washing the resulting pellet with buffer, which ensured the purity of the sample and maintenance of the viability of the bacteria in it.

RESULTS

278: It would be easier for readers if in table 5,6 and 7 is included a column with the OR *P-*value.

There is a reference of the P-value in the footnotes of the tables. We think that to include a new column with more numbers will not be useful for the readers. In addition, CI were estimated considering 95% confidence. Therefore, a CI that doesn´t include 1 is also indicative of a statistically significant result.

280: I wonder about the *P-*value of contact with other cats and dogs? The OR is close to or greater than 1.

Actually, the P-value for an unconditional model is not really relevant given that it is an unconditional probability and it is an intermediate result in building the conditional model. In addition to this, the OR is just a point estimation and it is also not relevant, the goal of all statistical analysis is to estimate the parameters of the population from the information obtained in the sample (the study).

309: as for urinary shedding (Table 6) ,P-value here is <0.05?

The same answer as for the previous question applies here.

DISCUSSION

350: Please reference the study to give the correct weight to the statement

Thank you for this comment. We totally agree and added more information about the study.

Now reads (Lines 356-358):

“Recently, a study from Malaysia demonstrated cultivation of pathogenic *Leptospira* spp. from renal tissue in 3/82 clinically healthy shelter cats and from both, renal tissue and urine, in 1/82 of these cats (Alashraf et al., 2020).”

352: What about cat’s urine pH?

The innate defense mechanisms of the host protect against invasion of bacteria, and the physicochemical properties of urine may be part of this defense system (Smee et al. 2013). Specifically, the potential mechanisms underlying antibacterial properties of urine include low pH and highly concentrated urine corresponding to high urine osmolality (Lees et al., 1979; Smee et al. 2013). However, Thornton et al (2018) experimentally demonstrated that *Escherichia coli* in vitro growth was higher in neutral to acidic and diluted urine compared to alkaline and concentrated urine. Leptospires do not survive well in acidic urine but remain viable in alkaline urine (Levett, 2001). In the present study, there was no significant difference in urine pH and urine specific gravity between PCR-positive (mean pH 6.7, mean USG 1.044) and PCR negative cats (mean urine pH 6.7, mean USG 1.040) (P>0.05), and between culture-positive (mean pH 6.3, mean USG 1.047) culture-negative cats (mean urine pH 6.7, mean USG 1.043) and (P>0.05). However, we have to be cautious in the sense that these were 220 culture-negative against 7 culture-positive individuals.

Lees GE, Osborne CA, Stevens JB. Urine: A medium for bacterial growth. Vet Clin North Am Small Anim Pract. 1979;9(4):611-6.

Smee N, Loyd K, Grauer G. UTIs in small animal patients: Part 1: Etiology and pathogenesis. J Am Anim Hosp Assoc 2013;49:1–7.

Thornton LA, Burchell RK, Burton, SE, Lopez-Villalobos N, Pereira D, MacEwan I, et al.; The Effect of Urine Concentration and pH on the Growth of Escherichia Coli in Canine Urine In Vitro. J Vet Intern Med 2018;32:752–6.

Levett PN. Leptospirosis. Clin. Microbiol. Rev. 2001,14(2):296. DOI: 10.1128/CMR.14.2.296-326.2001.

We included a new paragraph in the discussion and two new references. Please see new line 364-368:

“Low pH is another physicochemical property of the urine contributing to the defense system against bacteria (Smee et al., 2013), and *Leptospira* spp. do not survive well in acidic urine but remain viable in alkaline urine (Levett 2001). However, in the present study, the mean urine pH was identical in culture-positive and in culture-negative cats.”

Smee N, Loyd K, Grauer G. UTIs in small animal patients: Part 1: Etiology and pathogenesis. J Am Anim Hosp Assoc 2013;49:1–7.

Lees GE, Osborne CA, Stevens JB. Urine: A medium for bacterial growth. Vet Clin North Am Small Anim Pract. 1979;9(4):611-6.

371-372: This is a good place to explain and consider the demanding growing conditions for *L. interrogans* serovar Copenhageni.

We are sorry, but we do not understand the reason for this question, because, in the present study, *L. interrogans* serovar Copenhageni was the one that could be isolated indicating that it might grow easier than other serovars. We don´t have information on sequence types of the PCR-positive samples. But still, this indicates that other serovars might be more influenced by conditions of urine sampling/storage/processing in the laboratory and actually more difficult to grow in culture than *L. interrogans* serovar Copenhageni.

368: It would be interesting to discuss the *Leptospira* spp. found in these cats against those found in other studies in cats.

We discussed this aspect of the results and added respective references.

Please see new lines 396-402:

“Previously characterized *Leptospira* spp. isolates from cats´ kidney or urine had high similarity with *Leptospira interrogans* serovar Pomona (one cat from this area of Chile) [Ojeda et al., 2018] and *Leptospira interrogans* with high similarity to serovar Bataviae (4 cats from Malaysia) [Alashraf et al., 2020]. Whereas, *L. interrogans* Bataviae is reported to be the most common isolated serovar in humans, dogs and rats in Malaysia [Shaffei et al., 2012; Khor et al., 2016; Banacer et al., 2016), *L. interrogans* serovar Autumnalis is among the most commonly reported serovars in dogs and cats in Chile [Azocar-Aedo et al., 2014; Lelu et al., 2015; Azocar-Aedo and Monti 2015], and antibodies against *L. interrogans* Icterohaemorrhagiae have previously been reported in rodents in this area [Perret et al., 2005].”

Shafei MN, Sulong MR, Yaacob NA, Hassan H, Wan Mohamad WMZ, Daud A, et al. Seroprevalence of Leptospirosis among Town Service Workers in Northeastern State of Malaysia. Int J Collab Res Intern Med Public Health. 2012; 4(4):395–403.

Khor KH, Tan WX, Lau SF, Mohd AR, Rozanaliza R, Siti KB, et al. Seroprevalence and molecular detection of leptospirosis from a dog shelter. Trop Biomed. 2016; 33(2):276–284.

Benacer D, Mohd Zain SN, Sim SZ, Mohd Khalid MK, Galloway RL, Souris M, et al. t al. Determination of Leptospira borgpetersenii serovar Javanica and Leptospira interrogans serovar Bataviae as the persistent Leptospira serovars circulating in the urban rat populations in Peninsular Malaysia. Parasit Vectors. 2016; 9(1):1–11. doi: 10.1186/s13071-016-1400-1.

[Lelu](https://pubmed-ncbi-nlm-nih-gov.emedien.ub.uni-muenchen.de/?term=Lelu+M&cauthor_id=25880871) M, Muñoz-Zanzi C, [Higgins](https://pubmed-ncbi-nlm-nih-gov.emedien.ub.uni-muenchen.de/?term=Higgins+B&cauthor_id=25880871) B, [Galloway](https://pubmed-ncbi-nlm-nih-gov.emedien.ub.uni-muenchen.de/?term=Galloway+R&cauthor_id=25880871) R. Seroepidemiology of leptospirosis in dogs from rural and slum communities of Los Rios Region, Chile. BMC Vet Res. 2015; 11:31. doi: 10.1186/s12917-015-0341-9.

Perret PC, Abarca VK, Dabanch PJ, Solari GV, García CP, Carrasco LS, et al. Prevalencia y presencia de factores de riesgo de leptospirosis en una población de riesgo de la Región Metropolitana. Rev Méd Chile. 2005; 133:426-431. doi.org/10.4067/S0034-98872005000400005.

394-406: I agree with you. Considering that you have some clinical information about these cats, you can focus this part of the discussion on sick cats that shed leptospires, distinguish them as acute or chronic sick/infected, because those are two states of infection that are different.

We included this thought in the paragraph. It now reads (lines 427-437)

“These clinical signs could indeed be associated with leptospirosis and represent acute infection. In the other 10 sick cats with different organs affected, a causal relationship of infection with *Leptospira* spp. is not likely. The higher proportion of sick cats shedding pathogenic *Leptospira* spp. in the present study suggests that *Leptospira* spp*.* could be one of the etiological factors in the development of these clinical problems and that diagnostic testing should also be considered in cats with relevant clinical signs. Alternatively, it could be discussed that sick cats might be immunosuppressed and that an impaired immune status increases the risk of leptospiral infection and subsequent shedding. This, however, has not been reported so far. On the other hand, these cats could represent chronically infected individuals. It would have been interesting to further monitor these cats to see if they remained chronic shedders.”

408: Rats are reservoirs of the Icterohaemorrhagiae and Copenhageni serovars.

Six isolates of the present study were typed as *L. interrogans* serovar Copenhageni belonging to ST-17, which opens the interpretation about the source of infection. Taking into account that strains with this genotype in the PubMLST database have been isolated from rats, canines, porcine and humans (last accessed 19^th^ October 2018). In this regard, predation of infected hosts (mostly rodents) could also be a transmission route.

We added a sentence. Please see new lines 440-441

“In general, rodents are a natural reservoir pathogenic *Leptospira spp.*, and rats are reservoir hosts of the Icterohaemorrhagiae and Copenhageni serovars (Miraglia et al., 2013).”

Please include in the discussion information about why the positives to a single culture were not positives to IMS-culture and/or vice versa?

There are several possible reasons that explain the PCR negative results in a positive culture urine samples, but the main one should be due to inhibitor presences. In addition, the higher sensitivity of IMS-PCR than direct PCR and culture is explained based on the effectiveness of an IMS-system defined by its antigen-binding capacity or capture efficiency. Furthermore, although direct PCR loses sensitivity because of PCR inhibitors in urine samples, the IMS-qPCR can remove PCR inhibitors during the processing of clinical urine samples.

We added the following paragraph in the discussion. Please see new lines 374-376:

“One possible explanation for the discrepancy in the results of direct and IMS-coupled cultures is the hypothesis that antibodies used in IMS may have a neutralizing effect for the growth of *Leptospira* spp. in culture.”

**Reviewer #2:**

The purposes of this study were to determine the proportion of cats shedding pathogenic Leptospira in Southern Chile, to determine the molecular profile of the cultured isolates, and to identify possible risk factors regarding Leptospira spp. shedding.

I think the results of this paper are definitely very interesting and highlight the importance of further investigating leptospirosis in cats. However, there are still some issues need to be addressed before further consideration.

We thank the reviewer very much for this positive statement.

Abstract:

Line 25: Change the Leptospira to Leptospira spp.

We changed that accordingly throughout the document

Line 26: This sentence need to be rephrased as in the reference 19, it has been reported from urine and kidney.

We changed that accordingly. Please see new lines 24-26:

“Shedding of DNA of pathogenic *Leptospira* spp. has been documented in naturally infected cats in several countries, but urinary shedding of infectious *Leptospira* spp. has only recently been proven.”

Line 33: Immunomagnetic

We changed that accordingly

Introduction:

Line 59: should be under investigated rather than rarely suffer.

We agree that leptospirosis is probably underdiagnosed in cats but cats are also less susceptible to develop clinical disease as demonstrated by experimental infection by Larsson et al. (1985). We modified the sentence. It now reads (Line 58-59)

“Only few reports of clinical cases of leptospirosis in cats are published [7-9] and *Leptospira* spp. infection appears to be underinvestigated in cats compared with other animal species.”

Line 63: The proportions were also very much depending on samples size and laboratory techniques used. Prevalence is more commonly used. Suggest changing the proportions to prevalence.

Thank you for this comment. We changed proportions to prevalence.

Line 69-76: Suggest shortening the paragraph.

The paragraph has been modified. Now reads (Lines 73-76):

“Prevalence of cats shedding pathogenic *Leptospira* spp. in naturally infected cats has also been documented using urine PCR, varying from 0.8% in Thailand [14], 1.7% in Spain [15], 3.3% in Canada and Germany [11, 13], 4.9% in Malaysia [19], up to 67.8% in Taiwan [10]."

Line 84-86: This was the result obtained from previous publication, it will be more informative if authors can share about the importance of that finding.

Thank you for this comment.

We added one sentence. Please see new lines 86-87:

“This indicates that there is a considerable risk for infection for humans and mammalian animals living in this environment.”

Line 87: How big is the human population? Since this paper concerns about the role of cats in leptospirosis. Strongly suggest the authors to add in human leptospirosis in Chile in the introduction in order to highlight how severe is the problem in human and lead to the investigating in companion animal.

A paragraph including the human cases of leptospirosis and respective references were included.

Now reads (Lines 88-92):

"Leptospirosis has emerged to become a major public health threat in Latin America. Nevertheless, little is currently known regarding its incidence (WHO, 2003). In countries, such as Peru and Chile, where leptospirosis has been studied systematically in rural areas, a large proportion of the human population has antibodies, indicating previous exposure to pathogenic leptospires (36,6% and 12,0% respectively) (Silva-Díaz et al, 2015; Terrazas et al, 2012)."

Silva-Díaz, Heber , Dunalia N. Llatas-Cancino, Marcos J. Campos-Sánchez, Franklin R. Aguilar-Gamboa, Katya M. Mera-Villasis y Mayra Y. Valderrama-Ayén. 2015.Leptospirosis frequency and socio-demographic characteristics associated in febrile patients from northern Perú. Rev Chilena Infectol. 32 (5): 530-535.

Terrazas, Solana; Olea, Andrea; Riedemann, Stella and Torres, Marisa.2012. Prevalence of leptospirosis in adults in Chile. Rev Chilena Infectol. 2003, 29, 641-647.

Material and methods:

Line 104: Does the locations and time of sampling affected the result?

All samples were collected during spring and summer when environmental conditions are ideal for leptospires in this area due to the moderate temperature and high precipitations. We sampled in rural areas and urban areas. When considering previous studies (Azocar-Aedo et al., 2014), we do not expect much effect on the results.

Azocar-Aedo L, Monti G, Jara R. Leptospira spp. in Domestic Cats from Different Environments: Prevalence of Antibodies and Risk Factors Associated with the Seropositivity. Animals (Basel). 2014;4(4):612-626. doi: 10.3390/ani4040612.

Line 111: would suggest providing a justification for the inclusion for outdoor cat only in discussion part. The definition of outdoor need to be specified. Roam freely? Community cat?

We decided to include only cats with outdoor access as the risk for infection with because of the higher likelihood for exposure (hunting behaviour, drinking out of puddles) to *Leptospira* spp.. An outdoor cat was defined as a cat that has free access to outdoor and was not restricted to a balcony only. Most cats included in the study had owners and few cats were recently adopted stray cats. We specified this in a new paragraph in the material and methods section.

Now reads (Lines 119-127):

“Included cats were patients of the small animal hospital of the Universidad Austral de Chile in Valdivia presented for health issues or for elective procedures (e.g. neutering, vaccination), client-owned cats participating in castration projects offered by the city of Valdivia or other communities, and and cats presented by their owners specifically for study enrolement. Cats were offered one free vaccination and deworming as compensation for participating, if this was clinically justifiable. Cats were excluded from the study if they had received antimicrobial treatment within the last four weeks before sampling due to a decreased likelihood for detection of *Leptospira* spp. even if they were infected, or if they were living exclusively indoors or had only access to a balcony because of a low risk for infection.”

Line 117: The physical examination was performed by who? Was there any consent from owner?

All physical examinations were performed by the first author (RD) and the second author (JO). Informed written owner consent was obtained for all cats included in the study.

Line 124: MAT is always the gold standard in investigating leptospirosis. Is there any reason why it was not performed?

Thank you for this comment. And we agree that MAT is commonly performed to identify infection, however, as an antibody test, a single MAT could also indicate just exposure, therefore is not necessarily a good proxy of active infection and not of shedding, which was the aim of the study. Shedding of pathogenic *Leptospira* spp. is indicative of active infection, which provides a better indication of the zoonotic potential of that animal. Summarizing: Determination of MAT would not have provided additional information about shedding and thus, about the zoonotic risk.

Line 123: There is lack of justification the usage of qPCR, IMS-qPCR, culture and IMS-coupled culture concurrently in this study. From the line 336 to 346, IMS-qPCR was reported to be more sensitive than qPCR from the previous study. Hence, is the comparison between the different techniques also one of the objectives of the study?

The comparison between different techniques was not a goal of the study. The goal was to increase the diagnostic sensitivity by applying these four techniques. We removed the comparison of qPCR and IMS-coupled PCR from the results section and shortened the discussion accordingly.

Table 2

Please be more specific about the chronically ill. Would like to know what type of illness.

The chronically ill cats had the habitus of a chronically ill animal. This includes a reduced body condition score, dull fur and lethargy. Some of them also had ectoparasites (ear mites, fleas) and probably also endoparasites. Unfortunately, we were not able to perform a complete work-up of these cases as part of the study.

A footnote was included below Table 2, line 274:

*Cats had the habitus of a chronically ill animal, presenting with a reduced body condition score, dull fur and lethargy.

Discussion:

Line 332: The recruited cats were not indoor, hence, how accurate the owner can observe about the rat-hunting and eating behaviour?

Thank you for this comment. We agree with the reviewer that the owners of outdoor cats cannot completely be sure that a cat is not hunting despite they do not observe the cat doing it. However, owners often will find leftovers (fur, gallbladder) as cats typically don´t eat their prey completely. This allows the owner to make an assessment of the hunting behavior. Some owners (18) were not able to give an answer to this question and were therefore not included in the analysis.

Line 349-351: Since it has been reported before, I will recommend removing “first one”.

We rephrased the sentence: Now reads (Lines XX):

“In addition to one recent study from Malaysia [19] and one case report originating from the same geographic area as the present study, the present investigation as well yields positive *Leptospira* spp. culture results from feline urine.”

Line 355-356: Need to discuss a little bit about the findings obtained from direct culture and IMS-coupled culture. Why there were certain samples positive with direct culture, some with IMS-coupled culture?

Usually, culture isolation is difficult, and it has a low success rate. IMS could help to improve sensitivity because it concentrates the bacteria present in the sample, and probably these positive samples to IMS and negative to conventional culture are samples with low bacterial load and probably below the level for growth in direct culture or for competition with other organisms. In addition, due to the presence of many inhibitory substances, usage of magnetic separation enhances the analytical specificity and sensitivity of the subsequent detection method. There is, however also the possibility that the use of antibodies in the IMS have a neutralizing effect for the growth of Leptospira.

We added two sentences and one additional reference:

Now reads (Lines 369-371:

“Usage of magnetic separation enhances the analytical specificity and sensitivity of the subsequent detection method, given that it helps to remove the presence of many inhibitory substances or other organisms (Fernandes et al., 2008)."

Please see new lines 374-376:

“One possible explanation for the discrepancy in the results of direct and IMS-coupled cultures is the hypothesis that antibodies used in IMS could have a neutralizing effect for the growth of *Leptospira* spp. in culture.”

Line 424-425: it also affected by the stage pf the leptospirosis

We agree that shedding of Leptospira spp. is affected by the stage of disease.

We rephrased the sentence accordingly

Please see new line 462-464:

“Apart from the possible negative effect on bacterial viability of the hostile environment that is provided by feline urine and the stage of disease, there is another potential cause of negative culture result: the generally known difficulty to isolate *Leptospira* spp. from infected hosts and get them growing *in vitro*.”

Line 427: that should be one of the aims of the study

The comparison between different techniques was not a goal of the study. The goal was to increase the diagnostic sensitivity by applying these four techniques. We removed the comparison of qPCR and IMS-coupled PCR from the results section and shortened the discussion accordingly.
